# Postural stability after operative reconstruction of the AFTL in chronic ankle instability comparing three different surgical techniques

**DOI:** 10.1515/med-2024-1028

**Published:** 2024-09-02

**Authors:** Juergen Goetz, Clemens Baier, Georg Vitzethum, Joachim Grifka, Guenther Maderbacher, Hans-Robert Springorum

**Affiliations:** Department of Orthopaedic Surgery, Regensburg University Hospital, Regensburg, Germany

**Keywords:** ankle, ligament, repair, reconstruction, Level III, prospective, matched-pair comparative study

## Abstract

**Background:**

Chronic lateral ankle instability is a relatively frequent consequence after acute ankle sprain. In case of unsuccessful conservative treatment, surgical therapy is recommended to prevent osteoarthritis of the ankle joint. To date, different surgical methods have evolved. Yet, it remains unclear which approach reveals the best results. We hypothesized that the modified Broström–Gould procedure with suture anchor ligament fixation leads to superior postoperative results compared to the Broström–Gould procedure or the periosteal flap technique.

**Material and methods:**

In a prospective study, we examined the three surgical techniques. For this purpose, we performed a matched-pair analysis with four groups according to age, sex, and body mass index: periosteal flap technique (G1), Broström–Gould procedure (G2), modified Broström–Gould procedure with suture anchor ligament fixation (G3), and a control group (G4). Results were compared with the American Orthopaedic Foot & Ankle Society (AOFAS) score, a functional analysis as well as measuring postural stability with the Biodex balance system.

**Results:**

No significant differences were found between all four groups concerning AOFAS score, functional results, as well as postural stability.

**Conclusion:**

All three surgical methods revealed satisfactory results. No significant differences could be detected in clinical and functional categories. The Broström–Gould method as well as the modified procedure with anchor can be recommended as surgical therapy for chronic lateral ankle instability. Additional anchors do not seem to have a significant positive impact on the results.

## 1 Introduction

Acute sprains of the lateral ankle ligament are one of the most common injuries of the lower extremity. The lateral ligament complex consists of three different parts: the anterior talofibular ligament (ATFL), the calcaneofibular ligament (CFL), and the posterior talofibular ligament, the latter being the strongest of all three [1]. Regarding the classical supination trauma, the injury usually starts with the ATFL. Depending on the severity of the trauma, the CFL as the second part of the lateral ankle ligament follows. Injury to all three ligament parts is reserved for high-speed trauma. Apart from the injury of all three parts of the ligament, conservative treatment is recommended. For conservative/treatment, several protocols exist, including functional rehabilitation therapy, pharmacological anti-inflammatory drugs, and physiotherapy. Despite the success of nonoperative treatment, some patients do not respond, resulting in chronic ankle instability (CAI). CAI describes a lack of both functional and mechanical stability, resulting in pain, swelling, weakness, instability, and multiple episodes of the ankle “giving way” with persistent symptoms [2,3,4]. Finally, CAI can lead to end-stage osteoarthritis of the ankle. Concerning literature, it is the main source for posttraumatic ankle osteoarthritis [5].

According to previous studies, the prevalence of CAI in injured persons comprises 40% [6] to 70% [7]. If conservative treatment fails and satisfactory function and stability are not provided, operative treatment has to be considered [1].

Different techniques for reconstructing joint stability have been described in the literature. They are divided into anatomical and non-anatomical procedures. Anatomic reconstruction using the modified Broström–Gould technique seems to be the gold standard. The technique consists of shortening and stabilization of the damaged or weak ATFL, followed by mobilization of the inferior extensor retinaculum and suturing it to the distal fibula to reinforce it [8].

Another well-known technique uses a periosteal flap from the distal fibula to reinforce the damaged ATFL [9].

Extra-anatomical techniques use autologous or allogenic tendons grafts (e.g., peroneal tendons, achilles tendon, semitendinosus tendon) with extra-articular fixation (e.g., Watson-Jones) [10].

Newer techniques comprise arthroscopic reconstruction of the lateral ligaments, generally with the application of suture anchors. Respectively, intra-articular pathologies can be addressed as well [11,12].

In this study, three different procedures for the reconstruction of ankle stability were evaluated: Periosteal flap technique, Broström–Gould technique, and modified Broström–Gould technique using an additional suture anchor.

## 2 Materials and methods

### 2.1 Patients

This prospective study was designed to compare postural stability as well as functional ankle status after AFTL reconstruction with three subgroups:

Group 1 (G1): Periosteal flap technique.

Group 2 (G2): Broström–Gould technique.

Group 3 (G3): Broström–Gould technique with additional suture anchors.

An additional neutral group was built as the reference group (G4).

The participants were recruited at the Orthopaedic Dept. of the University Hospital of Regensburg, where they underwent surgery for reconstruction of ankle instability between January 2005 and December 2016.

Inclusion criteria were persistent ankle instability after conservative treatment of at least 6 months including orthosis and intense physiotherapy.

Exclusion criteria were patients older than 80 years of age, patients with neurologic disease (e.g., Parkinson), patients with ophthalmologic disorders, patients with vestibular deficiencies, patients with previous ankle fracture, previous ankle surgery, existence of total hip or knee arthroplasty, use of psychotropic drugs or medications that impair coordination, or patients not willing to participate.

The existence of any concomitant surgical procedures (e.g., tendon suture, cartilage-addressing measures) was exclusion criteria, too.

Group 1 consisted of 29 patients, group 2 consisted of 32 patients, and group 3 consisted of 24 patients to choose from. Group 1 consisted of 29, group 2 consisted of 32 patients, and group 3 consisted of 24 patients, from whom the selection could be made. All patients were matched due to gender, age, and body mass index (BMI). This resulted in a final group division for the matched patients. After inclusion, the final data set consisted then of eight patients in group 1, eight patients in group 2, and seven patients in group 3 (Table 1).

**Table 1:** Data of surgery and follow-up

	SG 1	SG 2	SG 3	*p*-value
Gender male	6	4	2	—
Gender female	2	4	5	—
Age at time of surgery (years)	33 (16;42;10.0)	33 (15; 48;12.3)	36 (17;56;15.4)	0.098
Duration of surgery (min)	(*n* = 7) 76 (56;96;14.4)	(*n* = 7) 49 (23;75;24.4)	(*n* = 4) 67 (34;105;29.7)	0.108
Surgical side left	2	1	5	—
Surgical side right	6	7	2	—
Time span from surgery to follow-up examination (months)	97 (18;126;38.0)	28 (5;38;11.2)	19 (6;37;12.0)	0.004
Age at follow-up (years)	43 (21;54;11.8)	36 (17;51;12.6)	37 (19;59;16.0)	0.730
BMI (kg/m^2^)	30 (26;36;3.5)	32 (23;50;9.3)	31 (23;37;5.1)	0.901

The number of the probands in the reference group (G4) was adapted to the number of patients in groups G1–G3: 23 healthy employees of the hospital and human medicine students formed the control group. Healthy probands were matched due to gender, age, and BMI. In total, 46 patients and probands were included in this study.

The indications for surgery were based on patient’s symptoms of persistent pain or ankle instability, with examination findings of local tenderness over the anterolateral aspect of the affected ankle, a positive anterior drawer test, or a positive talar tilt test. Conservative therapy failed after at least 6 months of treatment.

### 2.2 Surgical technique

#### 2.2.1 Group 1

The periosteal flap augmentation was performed with a 4–5 cm incision, from the ventrodistal tip of the fibula in the direction of the base of the fifth metatarsal. The intracapsular AFTL was exposed and incised and a part of the midportion was resected to adjust the tension of the ligament. Afterward, the incision was extended to proximal for about 2 cm to expose the lateral part of the fibula. A rectangular periosteal flap, measuring about 3 cm by 1 cm was detached from proximal to distal, the distal part of the flap still attached to the fibula. The inferior retinaculum was identified and mobilized. With the ankle in Pronation and slight dorsiflexion, the ligament was sutured. To reinforce the reconstructed ligament, the inferior retinaculum was sutured to the fibula. Then, the periosteal flap was flipped distally and sutured onto the distal insertion of the anterior fibulotalar ligament.

#### 2.2.2 Group 2

The Broström–Gould technique was performed by a 4–5 cm incision, from the ventrodistal tip of the fibula in the direction of the base of the 5th metatarsal. The intracapsular AFTL was exposed and incised and a part of the midportion was resected to adjust the tension of the ligament. The inferior retinaculum was identified and mobilized. With the ankle in Pronation and slight dorsiflexion, the ligament was sutured. To reinforce the reconstructed ligament the inferior retinaculum was sutured to the fibula.

#### 2.2.3 Group 3

The procedure was performed in the same way as described with group 2. To increase the strength, a suture anchor (Corkscrew 3.5, Arthrex^®^) was placed into the fibula using its FiberWires to attach the inferior retinaculum to the fibula. This procedure should guarantee a higher pull-out force and improve the tightness of the reconstructed AFTL.

In all cases post-operative aftercare included immobilization in a rigid orthosis with partial weight bearing for a period of 6 weeks, initially accompanied by lymphatic drainage and later with physiotherapeutic measures.

All operations were performed by three experienced foot and ankle surgeons.

### 2.3 Outcome assessment

Clinical outcome was assessed using the American Orthopaedic Foot & Ankle Society (AOFAS) ankle and hindfoot score (validated German version) [13].

The evaluation of the functional outcome also included the survey of anthropometric data, pre- and postoperative pain levels, subjective satisfaction, assessment of postoperative physiotherapy, and clinical examination using a standardized protocol.

Postural stability was measured by the Biodex Balance System (Biodex Inc. Shirley, NY, USA). This device consists of a multiaxial unstable, but gradually lockable platform, which is capable of tilting in the sagittal and transverse plane and a screen, located at head height. The platform measures and records the location of the center of mass of the person standing upright on the platform and displays it simultaneously on the screen. The maximum tilt of the platform is 20° in each direction. The apparatus prompts participants to center a cursor, viewed on a liquid crystal display, representing the center of mass while standing on the measuring platform. By altering the resistance of the platform to deviations, the level of difficulty for the patients can be altered (level 12 = locked platform, level 1 = most unstable setting). In our setting, two tests were performed with different levels of difficulty using levels 11 and 8.

**Figure 1:**
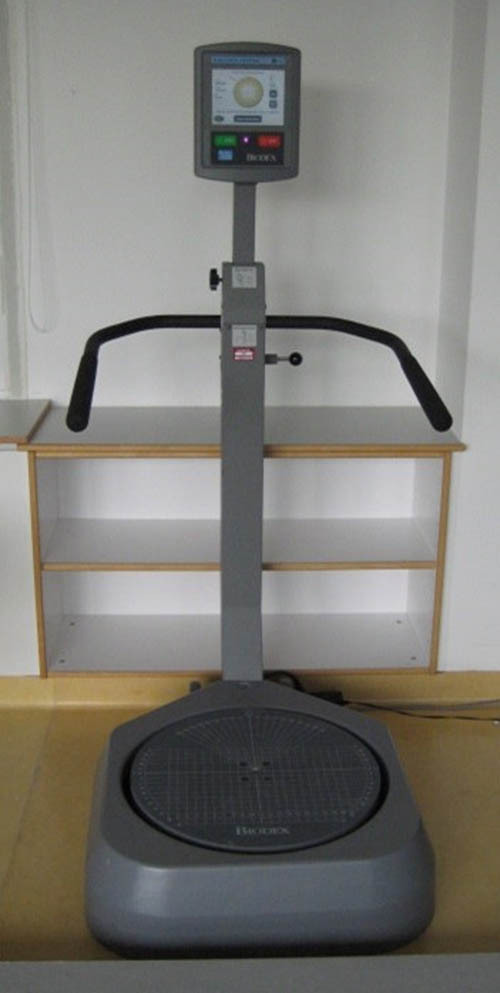
Biodex balance system.

**Figure 2:**
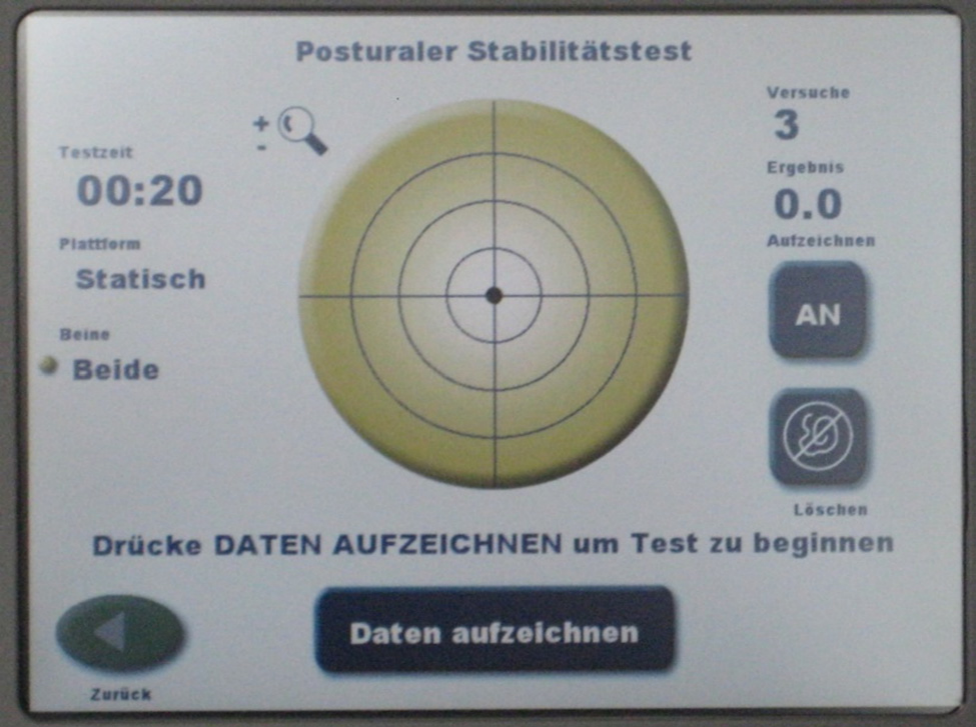
Screen capture of Biodex balance system.

The patients had to keep their center of mass (visible as a moving black dot on a screen in front of them) in the center of the target for 3 × 20 s. Patients and probands were positioned with legs hips-width apart and knees slightly bent. The hands were held over the handlebars for security reasons. However, all participants were instructed not to touch the handles unless they would otherwise fall off the stand. The participants had to stand on one leg and balance the black dot in the center for 3 × 20 s. We started with the operated leg. The standing leg was centered on the now locked platform and neither the other leg nor the hands were allowed to have contact with the system. The trial duration was set to 20 s and resting between trials was set to 10 s to prevent muscle tiredness. After the passage was completed, the opposite side was examined and, then, the examination was carried out in a two-legged stand. The test protocol as well as the examined parameters had been evaluated in other studies before [14,15]. In this study, we decided to repeat the procedure three times in total. In our experience, this setup is most likely to prevent the risk of falling, reduce the influence of muscle fatigue, and enable all patients to absolve this test (Figures 1 and 2).

The ability to balance is expressed by a balance index as a mean deviation in degrees of three required trials, calculated by using the time and deviation.

There are three different indices existing for postural stability: medial/lateral stability index (MLSI), anterior/posterior stability index (APSI), and overall stability index (OSI). MLSI and APSI represent only particular aspects of postural stability, which may be more interesting in, i.e., evaluating neurological deficits. While MLSI and APSI assess the fluctuations only in the mediolateral or anterior–posterior direction, respectively, the OSI as a composite of MLSI and APSI assesses the global status of postural stability concerning all directions. For data analysis, only the OSI was used to assess the global status of postural stability.

### 2.4 Statistics

Statistical analyses were performed using IBM SPSS Statistics Version 24.0 (IBM Corp., NY, USA). One-factor analysis of variance was used for interval-scaled and normally distributed characteristics in the comparison between subgroups. Independent samples *t*-test was used when comparing two groups on interval-scaled and normally distributed dependent characteristics. The dependent samples *t*-test was used for interval-scaled and normally distributed variables for comparison within a subgroup. For ordinal scaled or interval scaled and non-normally distributed data, the Wilcoxon test was carried out for comparison within a subgroup. The Mann–Whitney *U* test was used for comparison between the surgical subgroup and the associated control subgroup and the Kruskal–Wallis *H* test was used for comparison between the three subgroups. For the nominally scaled characteristics, the chi-square test was performed on cross-tabs. Variables were considered significant if *P*-values were less than 0.05. Patients excluded from the study for non-surgical associated reasons were not evaluated.


**Ethical approval:** The study protocol was approved by the local ethics committee (Nr. 17-430-101).
**Informed consent:** Written informed consent was obtained from all participants.

## 3 Results

### 3.1 Sample description

In total, 23 patients with surgical reconstruction after CAI were included in this study. Patients’ age at surgery was 34 years (12;56;12.1). Follow-up examination was 49 months after surgery (5;126;42.6). The mean BMI at follow-up was 31 kg/m^2^ (23;50;6.3). Subgroup data are listed in Table 1. Patients with reconstruction (group G1-3) and probands of the reference group (group G4) did not differ in gender, age, and BMI. Only the subgroup G1 did not show any significant differences concerning BMI (Table 2).

**Table 2:** OSI at level 11

Biodex balance system level 11				
Patients	Total	G1	G2	G3
Operated leg	1.6 ± 0.52	1.61 ± 0.57	1,46 ± 0.47	1.73 ± 0.54
Contralateral leg	1.41 ± 0.52	1.44 ± 0.52	1.46 ± 0.61	1.33 ± 0.51
Two-legged stance	1.00 ± 0.3	1.00 ± 0.27	1.00 ± 0.42	1.02 ± 0.23

Probands	Total	CG1	CG2	CG3
Two-legged stance	0.82 ± 0.28	0.83 ± 0.28	0.88 ± 0.32	0.76 ± 0.25

### 3.2 Complications

One patient of subgroup G1 showed impaired wound healing with MRSA superinfection, which healed without consequences after a wound revision. One patient in subgroup G3 suffered further supination trauma postoperatively and had to undergo revision surgery. Both patients were finally excluded from the study.

### 3.3 Functional evaluation

87% of all patients would undergo surgery once again (no significant difference in all subgroups). 56.5% of all patients stated that they had reached the preoperative activity level (no significant difference in all subgroups).

#### 3.3.1 VAS pain score

The average pre-operative pain NAS score (Table 1) was 6.3 and reduced to an average of 1.3 postoperatively at follow-up. Clinical examination did not show any significant differences in Range of motion and perceived pain in all subgroups. Within each subgroup, there was no significant difference between operated and non-operated legs. By clinical examination, no significant instability (lateral, medial, and drawer sign) could be detected in the subgroups

### 3.4 AOFAS (validated German version)

The calculated mean of the total (maximum 100 points) of the AOFAS score of all patients was 88.9 points. In the “periosteal flap” subgroup, the mean was highest at 91.8 points. The mean of the “Broström–Gould without anchor” subgroup was 88.3 points, and the mean of the “Broström–Gould with anchor” subgroup was 87.1 points. No statistically significant difference (*p* = 0.874) could be found between the three subgroups.

### 3.5 Biodex balance system (postural stability)

OSI of the operated leg at level 11 (very stable condition) was best in group G3; however, the significance level was missed. There was also no significant difference within the subgroups regarding OSI of operated and non-operated legs. No difference could be shown between the subgroups and the control group in the two-legged stance (Table 2).

At level 8 (more unstable condition), an almost identical result was presented (no significant difference between operated and non-operated leg in single stance and no significant difference in two-legged stance between each subgroup and the control group) (Table 3).

**Table 3:** OSI at level 8

Biodex balance system level 8				
Patients	Total	G1	G2	G3
Operated leg	1.45 ± 0.6	1.40 ± 0.66	1.30 ± 0.42	1.64 ± 0.71
Contralateral leg	1.55 ± 0.44	1.44 ± 0.43	1.44 ± 0.40	1.77 ± 0.45
Two-legged stance	1.07 ± 0.37	1.13 ± 0.31	1.03 ± 0.46	1.07 ± 0.36

Probands	Total	CG1	CG2	CG3
Two-legged stance	0.81 ± 0.28	0.89 ± 0.31	0.78 ± 0.28	0.76 ± 0.29

So, the study did not support the hypothesis that the modified Broström–Gould technique is superior to the Broström Gould technique or the periosteal-flap technique. There was no improvement in postural stability.

## 4 Discussion

CAI is a consequence of lateral ligament injuries that should not be underestimated. Different surgical procedures exist when conservative treatment fails. This study compared the functional and clinical outcomes of three different surgical techniques: the periosteal flap technique, the Broström–Gould procedure without using a suture anchor system and, as a third method, a modified Broström–Gould procedure using an additional suture anchor system. To our knowledge, no study has directly compared the three methods mentioned above to date.

Concerning biomechanical stability, Waldrop et al. compared the resilience of the AFTL in a biomechanical study using different surgical techniques [16]. Twenty-four post-mortem deep-frozen human ankle joints were randomly divided into four groups: an intact control group, a Broström group in which the method used by Broström 1966 was used, and two groups of suture anchor techniques (in the talus or tibia). They were able to prove that the biomechanical stability of the surgical procedures was equivalent but did not reach the load-bearing capacity of an intact AFTL. However, the study did not include the Broström–Gould procedure, where the extensor retinaculum is used to reinforce the sutured ligament.

Cho et al evaluated the 2-year outcome after using the Broström–Gould technique with transosseous suture, on the one hand, and the Broström–Gould technique with suture anchor systems, on the other hand. They did not find any significant differences between the two techniques [17].

Hu et al. [18] compared a modified Broström procedure using transosseous sutures with drill holes in the distal fibula and a modified Broström procedure with a suture anchor system. The postoperative results showed no significant differences between the compared surgical groups.

Mederake et al. [19] compared the results after arthroscopic modified Broström operation versus open reconstruction with local periosteal flap in CAI. Three months postoperatively, they found significantly better results concerning pain and instability in the arthroscopic group; 1 year postoperatively, the differences, however, were evened out.

Concerning our study, we found significant differences (*p* < 0.05) in all three surgical groups concerning pre- and postoperative pain. On average, the strongest pain postoperatively on the latest follow-up could be found in group 3 (the “Broström–Gould with anchor” group) (2.3 out of a maximum of 10 points). A possible reason for this might be the significantly different follow-up period, with the shortest follow-up in group 3 (*p* = 0.004). Respectively, the shortest recovery time comprised the “Broström–Gould with anchor” group (19 months (G3) vs 97 months (G1) or 28 months (G2)). The shorter recovery time could be a reason for the higher pain level at follow-up, as the adaptation and healing processes are probably not as advanced after 19 months as after 28 months, respectively, 97 months.

There were no statistically significant differences between the groups in the subjective assessment of postoperative stability; several studies have already proven that proprioception training has a positive effect on postural stability and thus leads to a stable feeling in the ankle joint [20]. This may have been the reason why we could not measure a significant difference between the groups in postural stability because all groups received proprioceptive training postoperatively.

Concerning pain scores after surgical lateral ligament stabilization, the literature shows comparable results. At the follow-up examination 12 months after Broström-Gould procedure the VAS pain score was found in between 1.2 and 1.8, respectively in between 1.2 and 2.12 24 months postoperatively [21,22].

Postural stability was evaluated using the Biodex Balance System. Neither the comparisons of postural stability between the operated and non-operated legs of the patients, nor the comparisons of the operated legs between the surgical groups, nor the comparisons between the surgical groups and the assigned control groups in the two-legged stance were found to be significant (*p* > 0.05). As Maeda et al. [23] were able to prove that CAI was associated with poorer dynamic postural control among adolescent athletes, this result has to be considered as a positive restoration of postural stability by the surgical reconstruction of the AFTL in all subgroups. Even more, unless Greve et al. [24] were able to determine a negative correlation between increased BMI and postural instability, the results of our study must be interpreted even more positive because although the BMI differed significantly, no significant difference in postural stability could be shown between the operation groups and the control group. Kim et al. [25] examined the postural stability of patients after anatomic ATFL repair augmented with suture-tape for CAI with the poor quality of remnant ligamentous tissue. In this study, a significant side-to-side difference concerning postural stability was found at follow-up ≥3 years. Comparing this result with our study, the restoration of autogenous ligament structure with its proprioceptive capacities could have a more positive influence on the recovery of postural stability than the substitution with allogenic material, e.g., suture-tape of fibre-wires in case of the absence of sufficient ligamentous tissue. Lee et al. reported deficits in proprioception and neuromuscular control 3 months after the modified Broström–Gould procedure in dynamic balance tests with the Biodex Stability system [26].

Concerning the AOFAS score, we could not detect any significant differences, with the “periosteal flap” group with the highest score of 91.8 points. As mentioned above, the time interval between surgery and follow-up was the longest in group 1 and could explain the slight differences. Also, Zeng was able to demonstrate the time-dependent improvement in the results 6 months, 1 year, and 2 years after lateral ligament reconstruction, using an InternalBrace™ [27]. Lee et al. [28] published a postoperative AOFAS score averaged 90.8 points after AFTL reconstruction, slightly higher than in our present study (88.3 points); Brodsky et al. [29] reported an AOFAS score of 95 points after Broström–Gould procedure. The follow-up period was 10.6 years respectively 5.3 years after surgical intervention indicating that the length of time after surgery should be considered as an influencing factor in outcome assessment.

## 5 Limitations

The small number of test subjects and the heterogeneous follow-up period must be critically assessed. Strongly reliable long-term results could not be demonstrated. Although the postoperative follow-up protocol was standardized, it was interpreted differently by the treating physiotherapists and therefore led to a bias.

Concerning different postoperative rehabilitation and different follow-up periods, our results must be interpreted carefully. Literature shows the influence of rehab protocols as well as follow-up periods after lateral ligament stabilization [30,31].

Furthermore, we did not include patients with arthroscopic lateral ligament stabilization. Numerous studies were published with this minimal-invasive approach. Reviews and meta-analysis, however, do not reveal a superiority of arthroscopic or open technique so far [32,33].

## 6 Conclusion

In conclusion, this study demonstrates that no one of the examined procedures is superior to the other. None of the results of the examinations carried out found a significant difference between the three compared surgical methods, which makes the three surgical methods appear to be equivalent. Likewise, the postoperative postural stability of the patients showed no significant difference to the postural stability of the healthy volunteers, which also demonstrated the success of the surgical procedure

## References

[1] Goru P, Talha S, Majeed H. Outcomes and return to sports following the ankle lateral ligament reconstruction in professional athletes: a systematic review of the literature. Indian J Orthop. 2021 Oct;56(2):208–15. 10.1007/s43465-021-00532-0, PMID: 35140851; PMCID: PMC8789970. PMC8789970 35140851

[2] Biz C, Nicoletti P, Tomasin M, Bragazzi NL, Di Rubbo G, Ruggieri P. Is kinesio taping effective for sport performance and ankle function of athletes with chronic ankle instability (CAI)? A systematic review and meta-analysis. Medicina (Kaunas). 2022 Apr;58(5):620. 10.3390/medicina58050620, PMID: 35630037; PMCID: PMC9146435. PMC9146435 35630037

[3] Cain MS, Ban RJ, Chen YP, Geil MD, Goerger BM, Linens SW. Four-week ankle-rehabilitation programs in adolescent athletes with chronic ankle instability. J Athl Train. 2020 Aug;55(8):801–10. 10.4085/1062-6050-41-19, PMID: 32577737; PMCID: PMC7462179. PMC7462179 32577737

[4] Herzog MM, Kerr ZY, Marshall SW, Wikstrom EA. Epidemiology of ankle sprains and chronic ankle instability. J Athl Train. 2019 Jun;54(6):603–10. 10.4085/1062-6050-447-17, Epub 2019 May 28 PMID: 31135209; PMCID: PMC6602402. PMC6602402 31135209

[5] Saltzman CL, Salamon ML, Blanchard GM, Huff T, Hayes A, Buckwalter JA, et al. Epidemiology of ankle arthritis: report of a consecutive series of 639 patients from a tertiary orthopaedic center. Iowa Orthop J. 2005;25:44–6; Valderrabano V, et al. Etiology of ankle osteoarthritis. Clin Orthop Rel Res. 2009;467:1800–6. doi: 10.1007/s11999-008-0543-6. PMC1888779 16089071

[6] Doherty C, Bleakley C, Hertel J, Caulfield B, Ryan J, Delahunt E. Recovery from a first-time lateral ankle sprain and the predictors of chronic ankle instability: a prospective cohort analysis. Am J Sports Med. 2016 Apr;44(4):995–1003. 10.1177/0363546516628870, Epub 2016 Feb 24. PMID: 26912285. 26912285

[7] Gribble PA, Bleakley CM, Caulfield BM, Docherty CL, Fourchet F, Fong DT, et al. Evidence review for the 2016 International Ankle Consortium consensus statement on the prevalence, impact and long-term consequences of lateral ankle sprains. Br J Sports Med. 2016 Dec;50(24):1496–505. 10.1136/bjsports-2016-096189, Epub 2016 Jun 3. PMID: 27259753. 27259753

[8] Tay KS, Chew CP, Lie DTT. Effect of periosteal flap augmentation on outcomes of modified Broström-Gould procedure for chronic lateral ankle instability. Foot Ankle Orthop. 2020 Jul 30;5(3):2473011420934735. 10.1177/2473011420934735, PMID: 35097395; PMCID: PMC8697196. PMC8697196 35097395

[9] Mittlmeier T, Rammelt S. Die Periostlappenplastik bei chronischer Instabilität des oberen Sprunggelenks [The periosteal flap augmentation technique in chronic lateral ankle instability]. Oper Orthop Traumatol. 2019 Jun;31(3):180–90. 10.1007/s00064-019-0600-1, Epub 2019 Apr 29. PMID: 31037329. 31037329

[10] Corte-Real N, Caetano J. Ankle and syndesmosis instability: consensus and controversies. EFORT Open Rev. 2021 Jun;6(6):420–31. 10.1302/2058-5241.6.210017, PMID: 34267932; PMCID: PMC8246108. PMC8246108 34267932

[11] Corte-Real NM, Moreira RM. Arthroscopic repair of chronic lateral ankle instability. Foot Ankle Int. 2009 Mar;30(3):213–7. 10.3113/FAI.2009.0213, PMID: 19321097. 19321097

[12] Guelfi M, Zamperetti M, Pantalone A, Usuelli FG, Salini V, Oliva XM. Open and arthroscopic lateral ligament repair for treatment of chronic ankle instability: A systematic review. Foot Ankle Surg. 2018 Feb;24(1):11–8. 10.1016/j.fas.2016.05.315, Epub 2016 May 12. PMID: 29413768. 29413768

[13] Kitaoka HB, Alexander IJ, Adelaar RS, Nunley JA, Myerson MS, Sanders M. Clinical rating systems for the ankle-hindfoot, mid-foot, hallux, and lesser toes. Foot Ankle Int. 1994;15(7):349–53. 10.1177/107110079401500701 7951968

[14] Arnold BL, Schmitz RJ. Examination of balance measures produced by the biodex stability system. J Athl Train. 1998;33(4):323–7. PMC1320582 16558529

[15] Woollacott MH, Tang PF. Balance control during walking in the older adult: research and its implications. Phys Ther. 1997;77:646–60. 10.1093/ptj/77.6.646 9184689

[16] Waldrop 3rd NE, Wijdicks CA, Jansson KS, LaPrade RF, Clanton TO. Anatomic suture anchor versus the Broström technique for anterior talofibular ligament repair: a biomechanical comparison. Am J Sports Med. 2012 Nov;40(11):2590–6. 10.1177/0363546512458420, Epub 2012 Sep 7. PMID: 22962291. 22962291

[17] Cho BK, Kim YM, Kim DS, Choi ES, Shon HC, Park KJ. Comparison between suture anchor and transosseous suture for the modified-Broström procedure. Foot Ankle Int. 2012 Jun;33(6):462–8. 10.3113/FAI.2012.0462, PMID: 22735317. 22735317

[18] Hu C-Y, Lee K-B, Song E-K, Kim M-S, Park K-S. Comparison of bone tunnel and suture anchor techniques in the modified Broström procedure for chronic lateral ankle instability. Am J Sports Med. 2013;41(8):1877–84. 10.1177/0363546513490647 23729687

[19] Mederake M, Hofmann UK, Ipach I. Arthroscopic modified Broström operation versus open reconstruction with local periosteal flap in chronic ankle instability. Arch Orthop Trauma Surg. 2022 Dec;142(12):3581–8. 10.1007/s00402-021-03949-2, Epub 2021 May 16 PMID: 33993359; PMCID: PMC9596524. PMC9596524 33993359

[20] McKeon PO, Ingersoll CD, Kerrigan DC, Saliba E, Bennett BC, Hertel J. Balance training improves function and postural control in those with chronic ankle instability. Med Sci Sports Exerc. 2008 Oct;40(10):1810–9. 10.1249/MSS.0b013e31817e0f92, PMID: 18799992. 18799992

[21] Wang AH, Su T, Jiang YF, Zhu YC, Jiao C, Hu YL, et al. Arthroscopic modified Broström procedure achieved similar favorable short term outcomes to open procedure for chronic lateral ankle instability cases with generalized joint laxity. Knee Surg Sports Traumatol Arthrosc. 2023 Sep;31(9):4043–51. 10.1007/s00167-023-07431-x, Epub 2023 May 10. PMID: 37162539. 37162539

[22] Woo BJ, Lai MC, Koo K. Arthroscopic versus open Broström-Gould repair for chronic ankle instability. Foot Ankle Int. 2020 Jun;41(6):647–53. 10.1177/1071100720914860, Epub 2020 Mar 24. PMID: 32207336. 32207336

[23] Maeda N, Ikuta Y, Tsutsumi S, Arima S, Ishihara H, Ushio K, et al. Relationship of chronic ankle instability with foot alignment and dynamic postural stability in adolescent competitive athletes. Orthop J Sports Med. 2023 Oct;11(10):23259671231202220. 10.1177/23259671231202220, PMID: 37859752; PMCID: PMC10583524. PMC10583524 37859752

[24] Greve J, Alonso A, Bordini ACPG, Camanho GL. Correlation between body mass index and postural balance. Clin (Sao Paulo). 2007;62(6):717–20. 10.1590/s1807-59322007000600010 18209913

[25] Kim SW, Cho BK, Kang C, Choi SM, Bang SM. Anatomic anterior talofibular ligament repair augmented with suture-tape for chronic ankle instability with poor quality of remnant ligamentous tissue. J Orthop Surg (Hong Kong). 2022 Sep-Dec;30(3):10225536221141477. 10.1177/10225536221141477, PMID: 36420544 36420544

[26] Lee JH, Jung HW, Jang WY. Proprioception and neuromuscular control at return to sport after ankle surgery with the modified Broström procedure. Sci Rep. 2022 Jan;12(1):610. 10.1038/s41598-021-04567-z, PMID: 35022508; PMCID: PMC8755731. PMC8755731 35022508

[27] Zeng GL, Cai LM, Xie Q, Huang HB, Li YC, Su BY. Anterior talofibular ligament all-inside arthroscopic reconstruction with InternalBrace™ for chronic lateral ankle instability. Med Sci Monit. 2022 Oct;28:e937699. 10.12659/MSM.937699, PMID: 36199231; PMCID: PMC9552571. PMC9552571 36199231

[28] Lee KT, Park YU, Kim JS, Kim JB, Kim KC, Kang SK. Long-term results after modified Brostrom procedure without calcaneofibular ligament reconstruction. Foot Ankle Int. 2011 Feb;32(2):153–7. 10.3113/FAI.2011.0153, PMID: 21288414. 21288414

[29] Brodsky AR, O’Malley MJ, Bohne WH, Deland JA, Kennedy JG. An analysis of outcome measures following the Broström-Gould procedure for chronic lateral ankle instability. Foot Ankle Int. 2005;26(10):816–9. 10.1177/107110070502601005 16221453

[30] Teramoto A, Murahashi Y, Takahashi K, Watanabe K, Yamashita T. Effect of accelerated rehabilitation on early return to sport after arthroscopic ankle lateral ligament repair. Orthop J Sports Med. 2022 Sep;10(9):23259671221121676. 10.1177/23259671221121676, PMID: 36119122; PMCID: PMC9478717. PMC9478717 36119122

[31] Zhao B, Sun Q, Xu X, Liu Y, Zhao Y, Gao Y, et al. Comparison of arthroscopic and open Brostrom-Gould surgery for chronic ankle instability: a systematic review and meta-analysis. J Orthop Surg Res. 2023 Nov;18(1):866. 10.1186/s13018-023-04292-5, PMID: 37964306; PMCID: PMC10644443. PMC10644443 37964306

[32] Wittig U, Hohenberger G, Ornig M, Schuh R, Leithner A, Holweg P. All-arthroscopic reconstruction of the anterior talofibular ligament is comparable to open reconstruction: a systematic review. EFORT Open Rev. 2022 Jan;7(1):3–12. 10.1530/EOR-21-0075, PMID: 35262506; PMCID: PMC8788150. PMC8788150 35262506

[33] Zhi X, Lv Z, Zhang C, Kong C, Wei S, Xu F. Does arthroscopic repair show superiority over open repair of lateral ankle ligament for chronic lateral ankle instability: a systematic review and meta-analysis. J Orthop Surg Res. 2020 Aug;15(1):355. 10.1186/s13018-020-01886-1, PMID: 32843055; PMCID: PMC7448467. PMC7448467 32843055

